# Chronic inflammation-induced senescence impairs immunomodulatory properties of synovial fluid mesenchymal stem cells in rheumatoid arthritis

**DOI:** 10.1186/s13287-021-02453-z

**Published:** 2021-09-14

**Authors:** Hyeon-Jeong Lee, Won-Jae Lee, Sun-Chul Hwang, Yongho Choe, Saetbyul Kim, Eunyeong Bok, Sangyeob Lee, Seung-Joon Kim, Hyun-Ok Kim, Sun-A Ock, Hae-Sook Noh, Gyu-Jin Rho, Sang-Il Lee, Sung-Lim Lee

**Affiliations:** 1 College of Veterinary Medicine, Gyeongsang National University, Jinju, 52828 Republic of Korea; 2 College of Veterinary Medicine, Kyungpook National University, Daegu, 41566 Republic of Korea; 3 Department of Orthopaedic Surgery, Gyeongsang National University School of Medicine and Hospital, Jinju, 52727 Republic of Korea; 4 Department of Internal Medicine and Institute of Health Sciences, Gyeongsang National University School of Medicine and Hospital, Jinju, 52727 Republic of Korea; 5 Animal Biotechnology Division, National Institute of Animal Science, Rural Development Administration, 1500 Kongjwipatjwi-ro, Isero-myeon, Wanju-gun, Jeollabuk-do 565-851 Republic of Korea; 6 Research Institute of Life Sciences, Gyeongsang National University, Jinju, 52828 Republic of Korea

**Keywords:** Mesenchymal stem cell-derived from the patient, Rheumatoid arthritis, Duration of inflammatory disease, Immunomodulation, Cellular senescence

## Abstract

**Background:**

Although the immunomodulatory properties of mesenchymal stem cells (MSCs) have been highlighted as a new therapy for autoimmune diseases, including rheumatoid arthritis (RA), the disease-specific characteristics of MSCs derived from elderly RA patients are not well understood.

**Methods:**

We established MSCs derived from synovial fluid (SF) from age-matched early (average duration of the disease: 1.7 years) and long-standing (average duration of the disease: 13.8 years) RA patients (E-/L-SF-MSCs) and then analyzed the MSC characteristics such as stemness, proliferation, cellular senescence, in vitro differentiation, and in vivo immunomodulatory properties.

**Results:**

The presence of MSC populations in the SF from RA patients was identified. We found that L-SF-MSCs exhibited impaired proliferation, intensified cellular senescence, reduced immunomodulatory properties, and attenuated anti-arthritic capacity in an RA animal model. In particular, E-SF-MSCs demonstrated cellular senescence progression and attenuated immunomodulatory properties similar to those of L-SF-MSC in an RA joint-mimetic milieu due to hypoxia and pro-inflammatory cytokine exposure. Due to a long-term exposure to the chronic inflammatory milieu, cellular senescence, attenuated immunomodulatory properties, and the loss of anti-arthritic potentials were more often identified in SF-MSCs in a long-term RA than early RA.

**Conclusion:**

We conclude that a chronic RA inflammatory milieu affects the MSC potential. Therefore, this work addresses the importance of understanding MSC characteristics during disease states prior to their application in patients.

**Graphical abstract:**

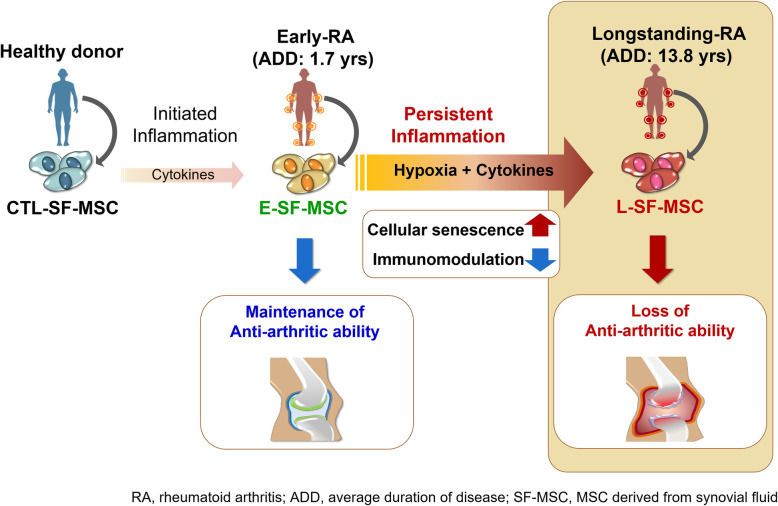

**Supplementary Information:**

The online version contains supplementary material available at 10.1186/s13287-021-02453-z.

## Background

The onset of rheumatoid arthritis (RA) is associated with loss of systemic immunological self-tolerance, which results in the activation of autoreactive immune cells that target collagen-rich joint regions and present symptoms characterized as chronic, destructive joint inflammation [1, 2]. As an autoimmune disease, there are limited curative options for RA that provide immunomodulation and articular cartilage or subchondral bone regeneration in the damaged joints. Mesenchymal stem cells (MSCs) can inhibit the immune response and are a potential candidate to treat various inflammatory autoimmune diseases, including RA [3]. Once MSCs are exposed to pro-inflammatory cytokines in an inflammatory milieu, they are activated and inhibit immune cell proliferation and activation by secreting anti-inflammatory cytokines [4, 5].

The utilization of MSC immunomodulation properties in RA patients is supported by the anti-arthritic potentials of MSCs observed in an RA animal model [3, 6]. However, there are some gaps in the in vivo studies, particularly regarding limited therapeutic effects, worsening of symptoms, and highly variable efficacy after MSC administration [6–8]. In addition, most of these studies were conducted using MSCs derived from mouse or non-patient specimens. Thus, investigating how RA affects MSCs derived from disease regions or MSCs transplanted into disease regions is required for clinical evaluation of potential therapeutic applications. Synovial fluid-derived MSCs (SF-MSCs) from RA patients are important for understanding disease pathogenesis and are easily obtained during the diagnosis or treatment of RA. Importantly, SF-MSCs have enhanced immunomodulation properties compared to bone marrow-derived MSCs (BM-MSCs) in collagen-induced arthritis (CIA) mouse models [3, 9]. However, the distinctive characteristics of SF-MSCs in an arthritis milieu are not well understood. Further, synovial fluid in an inflamed pathologic environment may result in alterations to the cells around inflammatory regions (genotoxic stress) and reduced ability to recover injuries [3, 10, 11].

Comparative studies of the immunomodulatory properties of activated MSCs from RA patients is a prerequisite for understanding the RA disease-affected cellular mechanisms of MSCs and how MSCs can be prepared for clinical applications in RA therapy. Given that SF-MSCs from RA patients (RA-SF-MSCs) have been exposed to the inflammatory milieu, we hypothesized that the progression of cellular senescence and the potential immunomodulatory properties are affected by pathological events and cellular environmental factors during RA. Therefore, we aimed to uncover alterations in duration-dependent immunomodulatory properties caused by inflammation-induced senescence in RA-SF-MSCs in an RA disease model.

## Materials and methods

### Chemicals used in experiments and ethical approval

All chemicals and media were purchased from ThermoFisher (Waltham, MA, USA) or Sigma-Aldrich Chemical Company (St. Louis, MO, USA), unless otherwise specified. Collection of synovial fluid (SF) specimens and PBMCs was performed after obtaining informed consent from patients and volunteers (Approval number GNUH 2012-05-009). The protocol for animal experiments in CIA mice was approved by the Animal Center for Biomedical Experimentation at Gyeongsang National University (GNU-131209-M068).

### Collection of SF and establishment of SF-MSCs from RA patients

Control SF was obtained from donors without evidence of inflammatory joint disease. SF from the RA groups was obtained from the joints of RA patients who were divided into early (E-RA, disease duration < 2 years) or long-standing (L-RA, disease duration > 10 years) groups. Then, the SF-MSCs were divided into three groups: CTL-SF-MSCs (*n* = 10), E-SF-MSCs (*n* = 9) and L-SF-MSCs (*n* = 12). The clinical histories of the RA patients are presented in Table 1. Cells were isolated from the aspirated and cultured SF and were processed as previously described [3]. The SF specimens were filtered through a 40-μm nylon cell strainer (BD Falcon, NJ, USA) to remove debris and centrifuged at 400×g for 10 min. The supernatants were stored at −80°C until the inflammatory cytokine analysis, while the cell pellet was resuspended and explanted onto 35 mm dishes (Nunc, Roskilde, Denmark). The cells were allowed to adhere for 2 days in culture medium before non-adherent cells were discarded. The adherent cells were cultured with advanced Dulbecco’s modified Eagle’s medium (ADMEM) supplemented with 10% fetal bovine serum (FBS), 1% GlutaMax, 10 ng/mL bFGF, and 1% penicillin and streptomycin (10,000 IU and 10,000 μg/mL) at 36.5°C in a humidified incubator with 5% CO_2_. The expanded cells were passaged four times before use for further analysis.

**Table 1:** Demographic and disease characteristics of donors

Characteristics	C-SF-MSCs (*n* = 10)	E-RA-SF-MSCs (*n* = 9)	L-RA-SF-MSCs (*n* = 12)	*P* value^*^
Age (years)	23.0 (1.22)	55.4 (14.1)	58.5 (8.6)	-
Women	3 (30%)	8 (88.8%)	12 (100%)	-
Positive for rheumatoid factor	-	8 (88.8%)	12 (100%)	-
Positive for anti-CCP antibody	-	8 (88.8%)	12 (100%)	-
DAS28-ESR	-	5.1 (1.4)	4.6 (1.1)	-
Disease duration (years)	-	1.7 (1.1)	13.8 (5.1)	0.011
Modified Sharp score	-	7.9 (8.8)	49.7 (26.4)	0.015

Data are means (SE) or n (%), ^*^P value indicates E-SF-MSC vs. L-SF-MSC. *C-SF* control synovial fluid, *ERA or LRA* early or long-standing rheumatoid arthritis, *MSC* mesenchymal stem cell, *CCP* cyclic citrullinated peptide, *DAS28-ESR* disease activity score 28-erythrocyte sediment rate

### Characterization of SF-MSCs

Expression of MSC-specific cell surface molecules in SF-MSCs were validated in triplicate by flow cytometry using a BD FACS Calibur instrument (BD Biosciences, NJ, USA). A total of 1 × 10^4^ cells were harvested and fixed with 4% paraformaldehyde at 4°C. All antibodies were diluted (1:200) with 1% bovine serum albumin (Table S1). Fluorescein isothiocyanate (FITC)-conjugated primary antibodies were incubated with the harvested cells for 1 h, with mouse IgG1-FITC used as an isotype control. Approximately ~80% of confluent SF-MSCs differentiated into adipocytes and osteoblasts after 3 weeks. Adipogenesis was induced with Dulbecco’s modified Eagle’s medium (DMEM) containing 10% FBS, 100 mM indomethacin, 10 mM insulin, and 1 mM dexamethasone. Then, adipogenesis was confirmed by intracellular lipid vacuole staining with 0.5% Oil red O solution and by gene expression (*FABP4* and *PPARγ*). Osteogenesis was induced with DMEM supplemented with 10% FBS, 200 mM ascorbic acid, 10 mM β-glycerophosphate, and 0.1 mM dexamethasone. Then, osteogenesis was determined by the accumulation of calcium deposits visualized with Alizarin-red S solution and by gene expression (*ON* and *OCN*). For chondrogenesis, 1 x 10^6^ SF-MSCs were cultured for 3 weeks in 15 mL tubes containing STEMPRO Osteocyte/Chondrocyte basal medium supplemented with 10% chondrogenesis supplement. Cell pellets were embedded in paraffin, cut into 5 mm sections, and stained with 1% Alcian blue and 0.1% nuclear fast red counterstain to confirm proteoglycan synthesis. Chondrogenesis was also verified by gene expression analysis (*COL2* and *COL10A1*). The protocol for the gene expression analysis is described below.

### Gene expression by quantitative PCR (qPCR)

qPCR was used for gene expression studies to determine pluripotency (*Oct3/4*, *Sox2*, and *Nanog*), apoptosis (*Bax*, *Bak*, *p53*, *Bcl2*, and *Birc*), differentiation (*FABP4*, *PPARγ*, *ON*, *OCN*, *COL2*, and *COL10A1*), and the expression of hypoxia-related genes (*GLUT1*, *LDHA*, *LOX*, and *PGK1*). Three replicates of each sample were analyzed by qPCR. Relevant primer information is displayed in Table S2. The total RNA was extracted using an RNeasy Minikit (Qiagen, CA, USA) and quantified using an OPTIZEN 3220 UV BIO spectrophotometer (Mecasys, Sungnam, Korea). Next, cDNA synthesis was performed from 1 μg total RNA using an Omniscript Reverse Transcription Kit (Qiagen) with a oligo dT primers at 60°C for 1 h. qRT-PCR was performed using a Rotor Gene Q qRT-PCR instrument (Qiagen) with Rotor-Gene 2× SYBR Green mix (Qiagen), 2 μL cDNA per reaction, and 0.5 mM forward and reverse primers. The qPCR program settings included of pre-denaturation (95°C for 10 min), 45 PCR cycles (95°C for 10 s, 60°C for 6s, and 72°C for 4 s), melting curve analysis (60°C to 95°C ramp, 1°C per seceond ramp rate) and cooling (40°C for 30 s). Transcript levels of all target genes were normalized against those of *TBP*, which is a stable reference gene in human MSCs [12].

### Proliferation and cell cycling in SF-MSCs

Vybrant MTT [3-(4,5-dimethylthiazol-2-yl)-2,5-diphenyltetrazolium bromide] Cell Proliferation Assays (Molecular Probes, Eugene, OR, USA) were used to evaluate SF-MSC proliferation. Absorbance at 540 nm was measured using a microplate reader (Molecular Devices). To analyze cell cycle changes, SF-MSCs were fixed with 70% ethanol, stained with 10 μg/ml propidium iodide (PI) solution, and evaluated using flow cytometry.

### Senescence-associated β-galactosidase activity staining

Cellular senescence was evaluated using Senescence β-Galactosidase Staining Kits (Cell Signaling Technology, Danvers, MA, USA). SF-MSCs were fixed for 15 min in fixation solution at room temperature, stained with β-Galactosidase staining solution, and incubated at 37°C overnight. To measure β-galactosidase activity, Mammalian β-Galactosidase Assay Kits (ThermoFisher, Rockford, IL, USA) were used. SF-MSCs were harvested, incubated with M-PER reagent for 10 min, and centrifuged for 10 min at 27,000xg. The supernatant was transferred into 96-well plates and treated with β-galactosidase reagent for 30 min at 37°C. The optical density was determined at 405 nm using a microplate reader (Molecular Devices).

### Evaluation of telomere length and telomerase activity

Telomere lengths in SF-MSCs were investigated using a nonradioactive chemiluminescent TeloTAGGG telomere restriction fragment (TRF) length assay kit (Roche, Indianapolis, IN, USA).

### Suppression of PBMC proliferation by SF-MSCs

Human PBMCs were isolated from healthy donors (*n* = 6) by density gradient centrifugation using Ficoll-Paque PLUS (GE Healthcare, Uppsala, Sweden). PBMCs were resuspended in RPMI 1640 complete medium supplemented with 10% FBS, 1% penicillin, and 1% streptomycin (10,000 IU and 10,000 μg/mL). Then, the cultures were stimulated with 1 μg/mL PHAL to activate T-cell proliferation. The PHAL-activated PBMCs (6.25 × 10^3^ cells/well) were seeded in a 96-well plates. After 12 h, 10 μg/mL Mitomycin-C (Sigma-Aldrich, USA) was added 2 h to inhibit cell proliferation. The PHAL-activated PBMCs were co-cultured for 5 days in a 96-well plate with pre-seeded SF-MSCs at PBMC to MSC ratios of 1:4, 1:2, and 1:1 before the addition of 5-bromo-2-deoxyuridine (BrdU). PBMC proliferation was evaluated using a Cell Proliferation ELISA, BrdU (colorimetric) Kit (Roche Diagnostics, Mannheim, Germany).

### Analysis of cytokine levels in SFs and SF-MSCs

The frozen SF supernatant samples were thawed and used to evaluate inflammatory cytokine levels. The levels of tumor necrosis factor (TNF)-α and interleukin (IL)-1β in the SF were determined with Quantikine ELISA kits (R&D Systems, Minneapolis, MN, USA). Briefly, standards and samples were incubated in wells pre-coated with the respective human primary antibody. The resulting antigen-antibody complexes were detected using human TNF-α or IL-1β conjugated to horseradish peroxidase, and the conjugate was quantified by a colorimetric reaction with 3,3′,5,5′-tetramethylbenzidine substrate. The resultant color intensity was read at 450 nm using a microplate reader (Molecular Devices). For SF-MSCs, 6.25 × 10^3^ cells/well were cultured in 96 well plates in serum-starvation medium (1% FBS in ADMEM), followed by supplementation with human recombinant TNF-α (50 ng/mL; R&D Systems) for 2 days to activate inflammatory cytokine production. After collecting the supernatant, the levels of matrix metalloproteinases (MMPs; MMP-1, MMP-3, and MMP-13) and other cytokines [IL-6 and indoleamine-pyrrole 2,3-dioxygenase (IDO)] were analyzed in the same manner as SF samples. All samples were assayed in duplicate and the concentration of target proteins in each sample was determined by interpolation from a standard curve.

### SF-MSC administration to CIA mice

Injection of MSCs into CIA mice was conducted as previously described [3]. Briefly, pathogen-free male DBA/1 mice (7–9 weeks old; Orient Bio, Seoul, Korea) were immunized with 100 μg bovine type II collagen (Chondrex, Redmond, WA, USA) emulsified in complete Freund’s adjuvant (CFA, Chondrex) by injection into the intradermal region of the tail on day 0. Mice received a booster immunization of an equal volume of bovine type II collagen and incomplete Freund’s adjuvant (IFA, Chondrex) on day 21. The experiment included 4 groups (*n* = 8 per group): a PBS injection control, and CTL-SF-MSC-, E-SF-MSC-, or L-SF-MSC-injected groups. SF-MSCs were intraperitoneally injected on day 21 and for five consecutive days with 200 μL PBS or SF-MSCs (5 × 10^6^ cells in 200 μL PBS). Clinical arthritis scores (0–4 scale) were evaluated for each limb in accordance with a well-defined standard. The total possible score was 16. To measure hind paw thickness, a caliper was placed across the ankle joint at the widest point. On day 48, CIA mice were sacrificed by cervical dislocation. The hind paws were scanned with a SkyScan 1076 micro-CT apparatus (Bruker, Kontich, Belgium) and reconstructed into a three-dimensional structure with a voxel size of 18 μm using NRecon and CT Analyzer software (Bruker). Joint tissue specimens from CIA mice were fixed with 10% formalin, decalcified for 3–4 weeks in 10% EDTA, and embedded in a paraffin block. Joint sections (5 μm) were stained with hematoxylin and eosin (H&E), Safranin O, or tartrate-resistant acid phosphatase (TRAP) to evaluate articular inflammation, cartilage damage, and TRAP-positive multinucleated cells (osteoclasts), respectively. The total number of TRAP-positive multinucleated cells containing three or more nuclei was counted in 10 areas of each CIA mouse ankle [3, 13].

### Induction of the RA-like inflammatory milieu

Because both low partial oxygen pressure (hypoxia) and inflammation are relevant features in the synovial joints of RA patients [14], an in vitro RA-like inflammation milieu was induced in E-SF-MSCs to explore whether immunomodulatory properties and senescence were altered in inflammation-exposed SF-MSCs. E-SF-MSCs were cultured in normal culture conditions for 3 days with in various gas compositions: 21% O_2_, 5% CO_2_, and 74% N_2_; or 3% O_2_, 5% CO_2_, and 92% N_2_ in a 95% humidified atmosphere. The cells were maintained in multi-gas incubators (ASTEC, Fukuoka, Japan) to reflect normoxia or hypoxia. In addition, the media for hypoxic E-SF-MSCs was supplemented with 20 ng/mL TNF-α and 20 ng/mL IL-1β (R&D Systems) as representative inflammatory cytokines. Both E-SF-MSCs and L-SF-MSCs cultured in normoxic conditions were used as controls.

### Western blot analysis

The induction of hypoxia was validated by upregulated hypoxia-inducible factor 1-α (HIF1α) expression. Cell extracts of E-SF-MSCs with or without hypoxia were prepared with RIPA buffer supplemented with Halt Protease Inhibitor Cocktail (Pierce Biotechnology, Rockford, IL, USA). The total protein concentration in the cell extracts was quantified using Bicinchoninic Acid Protein Assay Reagent Kits (Pierce Biotechnology). A 25-μg aliquot from each sample was fractionated by 10% SDS-PAGE and was transferred onto a polyvinylidene difluoride membrane (Millipore, Darmstadt, Germany). The membranes were blocked with 0.1% bovine serum albumin (BSA), incubated with anti-HIF-1α or anti-GAPDH primary antibodies (1:100 dilution with BSA) at 4°C overnight, incubated with horseradish peroxidase-conjugated secondary antibodies (1:3,000 dilution with BSA) at RT for 1 h, and detected using a chemiluminescence assay (Amersham Biosciences Corp, Piscataway, NJ, USA) with X-ray film for visualization.

### Apoptosis assays

The proportion of apoptosis in E-SF-MSCs with or without the induction of an RA-like inflammatory milieu was determined using an Annexin V-FITC Apoptosis Detection Kit (Invitrogen, Eugene, OR, USA). For these assays, 1 × 10^4^ cells were harvested, washed twice in PBS, and resuspended in 200 μL binding buffer. Then, the cells were treated with 10 μL Annexin V stock solution, incubated at 4°C for 30 min, counterstained with PI, and analyzed by flow cytometry (BD FACS Calibur).

### IDO activity measurements

IDO activity was measured as previously described [15]. Cultures were harvested and 2.5 × 10^4^ E-SF-MSCs with or without induction of an RA-like inflammatory milieu were cultured for 4 days. Then, the cells were supplemented with 100 μM L-tryptophan (Sigma) for 4 h. The supernatant was harvested and mixed with 30% trichloroacetic acid (Sigma) before an additional incubation at 50°C for 30 min. This solution was diluted in Ehrlich reagent (1:1) (Sigma, USA), and the optical density was measured at 492 nm using a microplate reader (Molecular Devices). Serially-diluted l-kynurenine (Sigma) made with fresh culture medium was used as the standard.

### Statistical analysis

Statistical significance was analyzed using paired T tests, one-way analysis of variance (ANOVA), and Tukey’s multiple comparison tests followed by Games-Howell post hoc analysis using SPSS 21.0 (IBM, Armonk, NY. USA). All data are presented as mean ± standard deviation (SD). *P* < 0.05 was considered significantly different.

## Results

### RA-SF-MSCs exhibit a normal phenotype and differentiation regardless of disease status

**Fig. 1:**
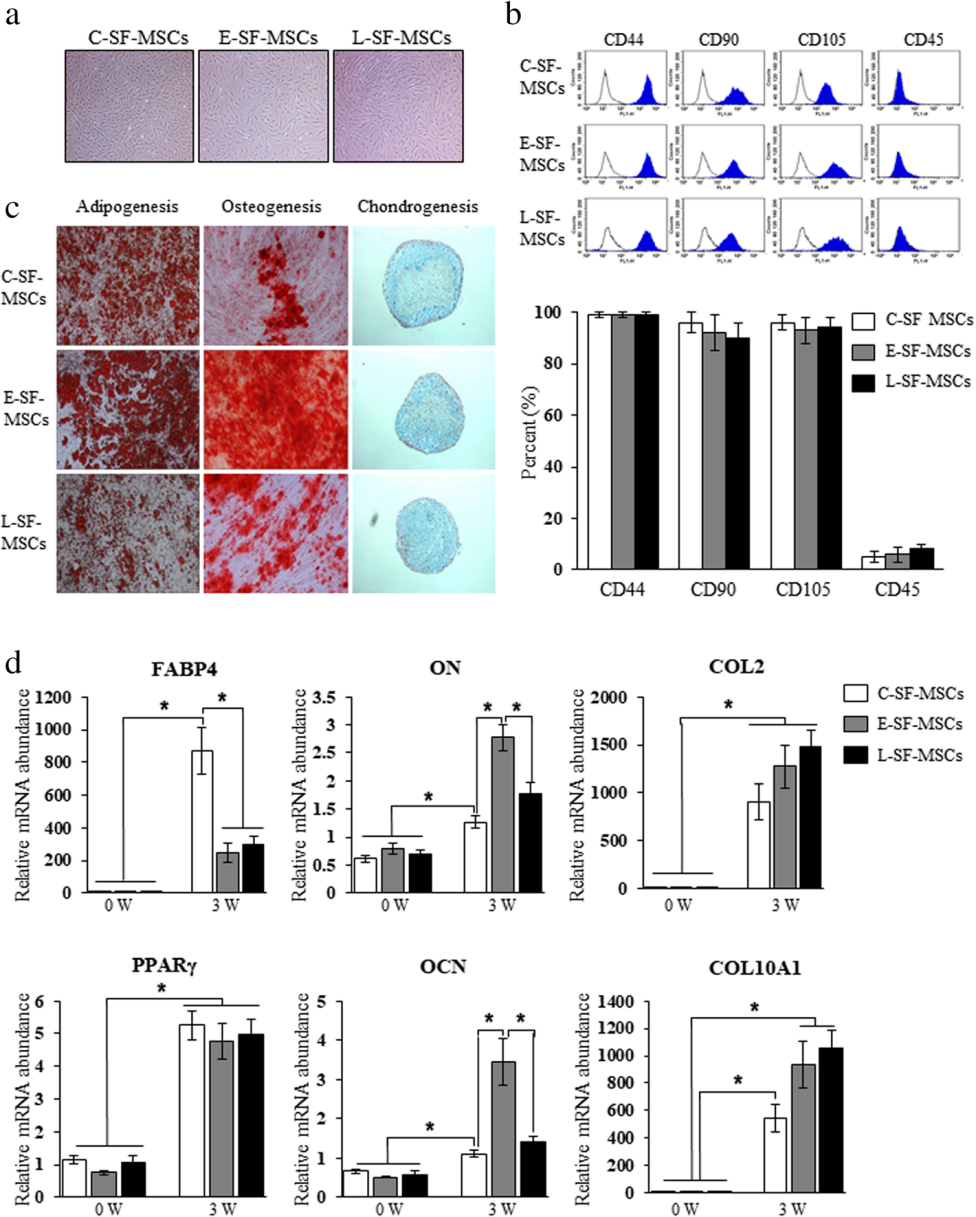
Characteristics and differentiation abilities of RA patient-derived SF-MSCs depend on disease duration. **a** A plastic-adherent population with fibroblastic morphology. **b** Flow cytometry indicates the positive expression of MSC-specific molecules (CD44, CD90, and CD105) and the negative expression of a hematopoietic cell surface molecule (CD45) within this SF-MSC population. **c** Cytochemical staining of differentiated SF-MSCs showing adipocytes (lipid droplets), osteoblasts (calcium deposits), and chondrocytes (proteoglycan synthesis) when grown in vitro and **d** changes in lineage-specific gene expression. The asterisks indicate significant differences. The data represent percentage mean values ± SD. 40× magnification. 0W, undifferentiated SF-MSC at the start of the culture; 4W, differentiated SF-MSCs for 4 W

RA patients were classified as having early (E-RA) or long-standing (L-RA) RA according to disease duration and the degree of joint destruction. Thus, L-RA patients had a longer disease duration and more severe joint destruction (modified sharp score) than E-RA patients (Table 1). Three types of SF-MSCs were derived from healthy controls (CTL-SF-MSCs, *n* = 10), E-RA patients (E-SF-MSCs, *n* = 9), and L-RA patients (L-SF-MSCs, *n* = 12). All SF-MSCs presented normal MSC phenotypes, such as plastic-adherent populations with fibroblastic morphology and positive MSC-specific or -negative hematopoietic-specific cell surface molecule expression (Fig. 1a, b). In specific cytochemical staining, adipogenic- and osteogenic-differentiated SF-MSCs showed strong positive stains, as confirmed by the cytoplasmic accumulation of lipid vacuoles and the deposition of calcified extracellular matrix by Oil red O and Alizarin-red S staining, respectively. When induced toward chondrogenic differentiation, all SF-MSCs were successfully aggregated were positively stained with Alcian blue. The staining intensity did not differ among the differentiated SF-MSCs upon gross observation (Fig. 1c). Adipogenesis-, osteogenesis-, and chondrogenesis-related genes in differentiated SF-MSCs were analyzed before and after the induction of differentiation. Adipocyte-related genes (*FABP4* and *PPARγ*) were significantly increased (*P* < 0.05) after differentiation into adipocytes compared to the undifferentiated state, but the levels of *FABP4* in E- and L-RA-SF-MSCs (246.1±1.1 and 294.5±1.1) were significantly (*P* < 0.05) lower than in C-SF-MSCs (868.5±0.9). After differentiation, the levels of osteocyte (*ON* and *OCN*)- and chondrocyte (*COL2* and *COL10A1*)-related genes in SF-MSCs were significantly increased (*P* < 0.05) compared to undifferentiated cells (Fig. 1d). Though there were slight differences in degrees, all SF-MSCs exhibited the ability to differentiate into multiple mesenchymal lineages such adipocytes, osteoblasts, and chondrocytes, with significantly increased (*P* < 0.05) lineage-specific gene expression. Therefore, the differentiation ability of E- and L-SF-MSCs did not decrease compared to C-SF-MSCs (Fig. 1c, d). Overall, we conclude that the phenotype and differentiation ability of SF-MSCs were not reduced by the RA disease status.

### Impaired proliferation and increased senescence in L-SF-MSCs

**Fig. 2:**
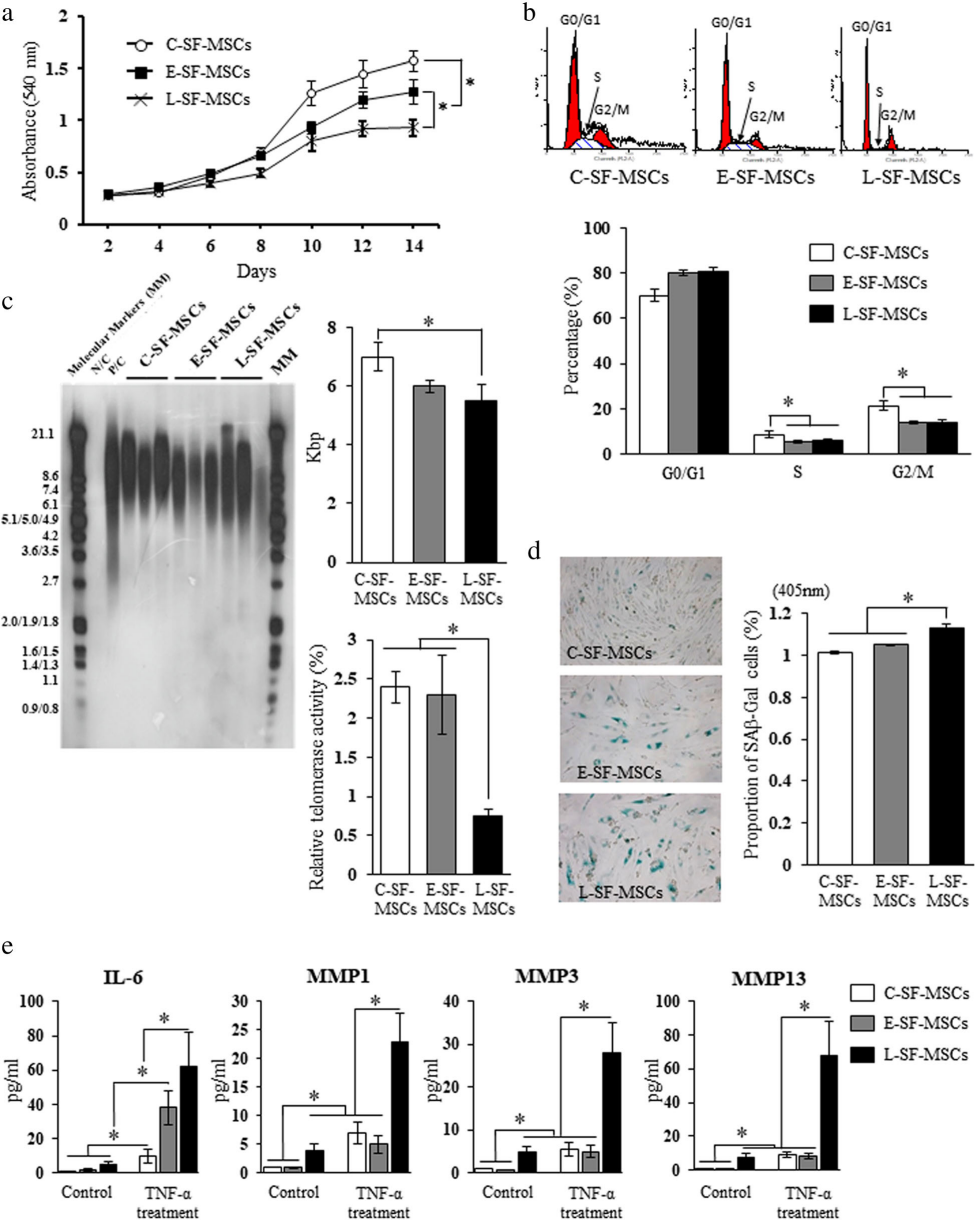
Proliferative ability and cellular senescence of RA patient-derived SF-MSCs. **a** Comparison of SF-MSC proliferation by MTT assay. **b** Cell cycle status of SF-MSCs by flow cytometry. **c** The range of telomere restriction fragment (TRF) lengths (kb) in SF-MSCs by non-radioactive chemiluminescent assay. **d** Evaluation of the relative telomerase activity (RTA) in SF-MSCs by qRT-PCR. **e** Comparison of β-galactosidase-positive cell populations in SF-MSCs. 100× magnification. The asterisks indicate significant differences. The data represent mean values ± SD. N/C, negative control; P/C, positive control; SA β-gal, senescence-associated β-galactosidase; SASP, senescence associated secretory phenotype; MMPs, matrix metalloproteinases

RA-SF-MSCs had significantly lower (*P* < 0.05) proliferation ability with a slower cell cycle than CTL-SF-MSCs (Fig. 2a, b). Telomere shortening, inactivation of telomerase, and increased β-galactosidase activity (β-gal) were significantly increased (*P* < 0.05) in L-SF-MSCs, indicating altered cellular senescence patterns (Fig. 2c, d). Moreover, inflammatory cytokine-treated L-SF-MSCs had a significantly higher (*P* < 0.05) senescence-associated secretory phenotype (SASP) than CTL-SF-MSCs and E-SF-MSCs (Fig. 2e). In E- and L-SF-MSCs, the expression level of *Oct3/4* and *Sox2* was not differ but *Nanog* (0.55 ± 0.05 and 0.51 ± 0.07), a pluripotent factor in stem cells was significantly lower (*P* < 0.05) than CTL-SF-MSCs (1.0 ± 0.11) (Fig. 6). The apoptosis-related factors, the expression levels of *Bax* and *Bcl-2* in E-and L-SF-MSCs (1.21 ± 0.05 and 0.76 ± 0.04) was significantly (*P* < 0.05) higher and lower than in CTL-SF-MSCs (1.17 ± 0.09 and 0.78 ± 0.05). The expression levels of *Bak* and *Birc* in L-SF-MSCs (1.01 ± 0.07 and 0.7 ± 0.08) was significantly (*P* < 0.05) higher and lower than in E- (0.75 ± 0.03 and 2.55 ± 0.5) and CTL-SF-MSCs (0.64 ± 0.03 and 2.40 ± 0.3), respectively. However, expression level of *p53* did not differ between all groups. Collectively, these results suggest that L-SF-MSCs are more senescent than both CTL-SF-MSCs and E-SF-MSCs.

### Reduced in vitro immunomodulatory properties of L-SF-MSCs

**Fig. 3:**
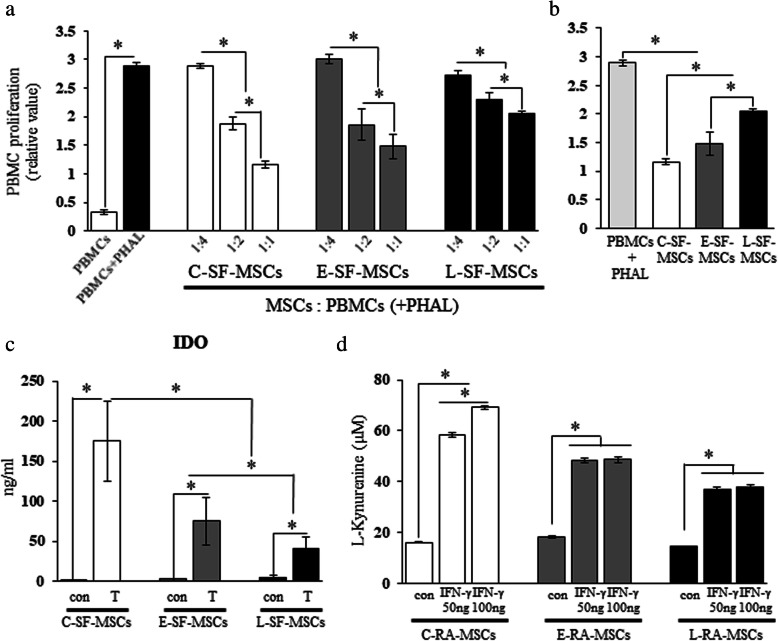
In vitro immunomodulatory properties of RA patient-derived SF-MSCs. **a** Evaluation of inhibited PBMC proliferation. The ratios 1:4, 1:2, and 1:1 indicate the cell population of pre-seeded SF-MSCs and PBMCs. PBMC and PBMC+PHAL indicate PBMC cultures without or with PHAL, respectively. **b** Comparison of inhibited PBMC proliferation in the highest PBMC to MSC ratio (1:1). **c** Quantification of IDO secretion after treatment with inflammatory cytokines by ELISA. Con or T indicate cells that were untreated or treated with inflammatory cytokines, respectively. **d** Evaluation of IDO activity after treatment with IFN-γ by measurement of L-kynurenine. The asterisks indicate significant differences. The data represent mean values ± SD. PBMC, peripheral blood mononuclear cells; PHAL, phytohemagglutinin-L; IDO, indoleamine 2,3-dioxygenase; IFN-γ, interferon-γ

Although all SF-MSC types suppressed PBMC proliferation (Fig. 3a), the intensity of this suppression varied depending on the group (Fig. 3b). Compared to the control group (PBMC + PHAL), the suppression of PBMC proliferation (PBMC to MSC = 1:1) of CTL-SF-MSCs and L-SF-MSCs was significantly (*P* < 0.05) higher and lower, respectively. In addition, IDO secretion in L-SF-MSCs post-TNF-α treatment (40 ± 15) did not reach that of CTL-SF-MSCs (175 ± 50) (Fig. 3c). Furthermore, IDO activity in L-SF-MSC after 50 ng and 100 ng IFN-γ treatment (36.72 ± 1.08 and 37.91 ± 0.71) significantly decreased (*P* < 0.05) more than that of CTL-SF-MSC (58.41 ± 0.77 and 69.77 ± 0.62) (Fig. 3d). Overall, the in vitro immunomodulatory properties of L-SF-MSCs were attenuated more than in other SF-MSCs.

### Attenuation of the anti-arthritic properties of L-SF-MSCs in CIA mice

**Fig. 4:**
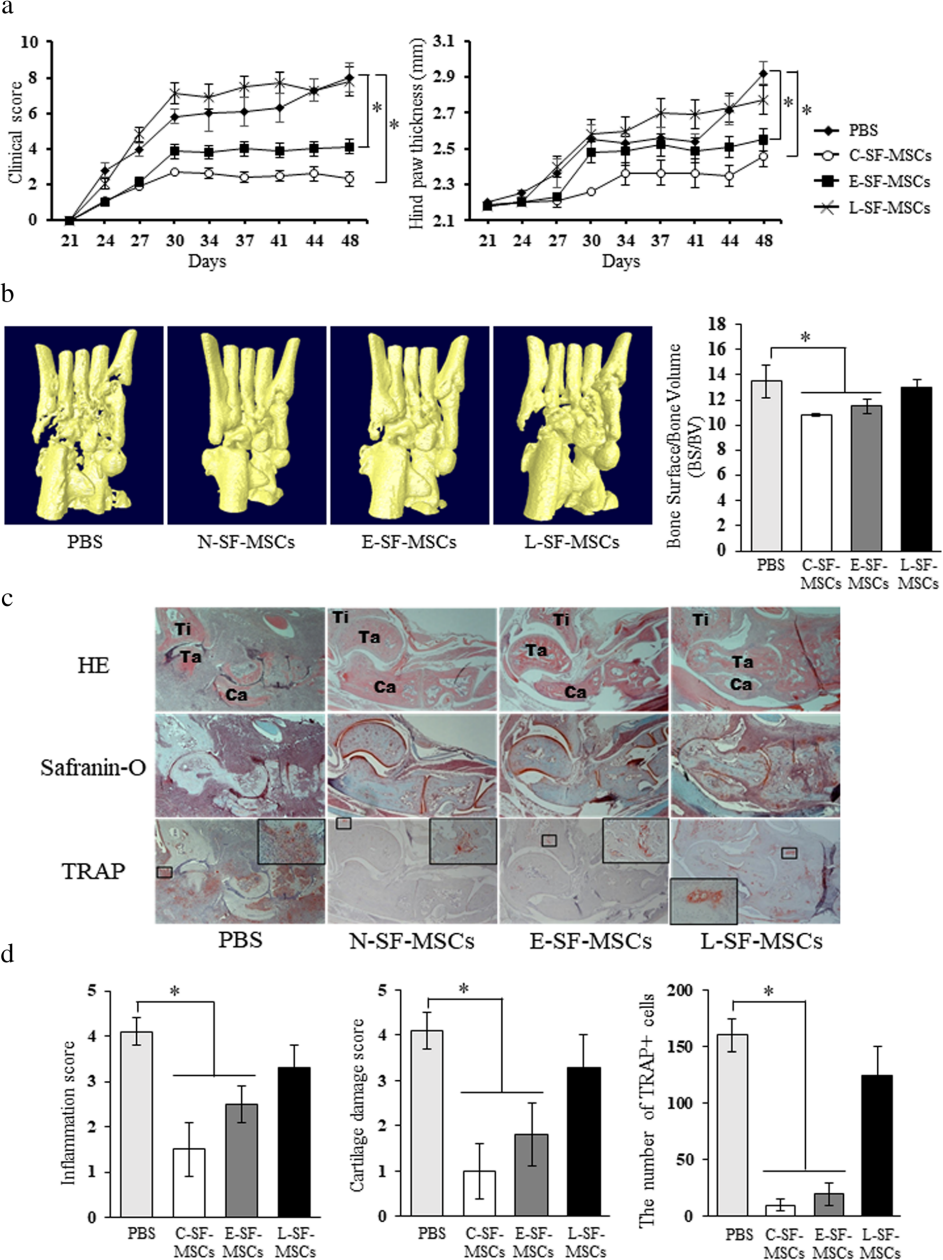
In vivo anti-arthritic potential of RA patient-derived SF-MSCs. **a** Evaluation of the clinical scores and measurements of hind paw thickness in CIA mice after intraperitoneal administration of SF-MSCs or PBS. **b** Images of micro-CT scanning and bone surface/bone volume (BS/BV) analysis. **c** and **d** Histological examination of therapeutic effects of SF-MSCs. In the HE staining, Ti represents tibia, Ta represents talus, and Ca represents calcaneus as the anatomical locations. 40× or 200× magnification. Graphs summarize the pathological scores in terms of inflammation, cartilage damage, and TRAP + cell (osteoclast) populations in joints from CIA mice. The asterisks indicate significant differences. The data represent the mean values ± SD. PBS, phosphate-buffered saline; H&E, hematoxylin and eosin; TRAP, tartrate resistant acid phosphatase. In the HE staining (panel **c**), Ti represents tibia, Ta represents talus, and Ca represents calcaneus as the anatomical locations

A significantly lower (*P* < 0.05) mean arthritis score and ankle thickness of CIA mice injected with CTL- and E-SF-MSCs indicated the therapeutic effects of SF-MSCs. However, L-SF-MSCs demonstrated no therapeutic potential and had worse symptoms 44 d post-MSCs application (Fig. 4a). Bone destruction did not progress in CTL- and E-SF-MSC-injected CIA mice, but was detected in the joints of L-SF-MSC-injected mice (Fig. 4b). Histopathological observation of the joints of CIA mice post-CTL- and E-SF-MSC application demonstrated arthritis prevention, mice injected with L-SF-MSCs presented arthritis-related changes such as synovial inflammation, cartilage damage, and osteoclast activity (Fig. 4c). Based on these in vitro and in vivo results, we conclude that the preventative effects of SF-MSCs are varied and depend on disease duration. Furthermore, L-SF-MSCs lost most of their immunomodulatory properties, possibly due to prolonged exposure to the RA-associated inflammatory milieu.

### Cellular alteration of E-SF-MSCs into L-SF-MSCs in the RA-mimetic milieu

**Fig. 5:**
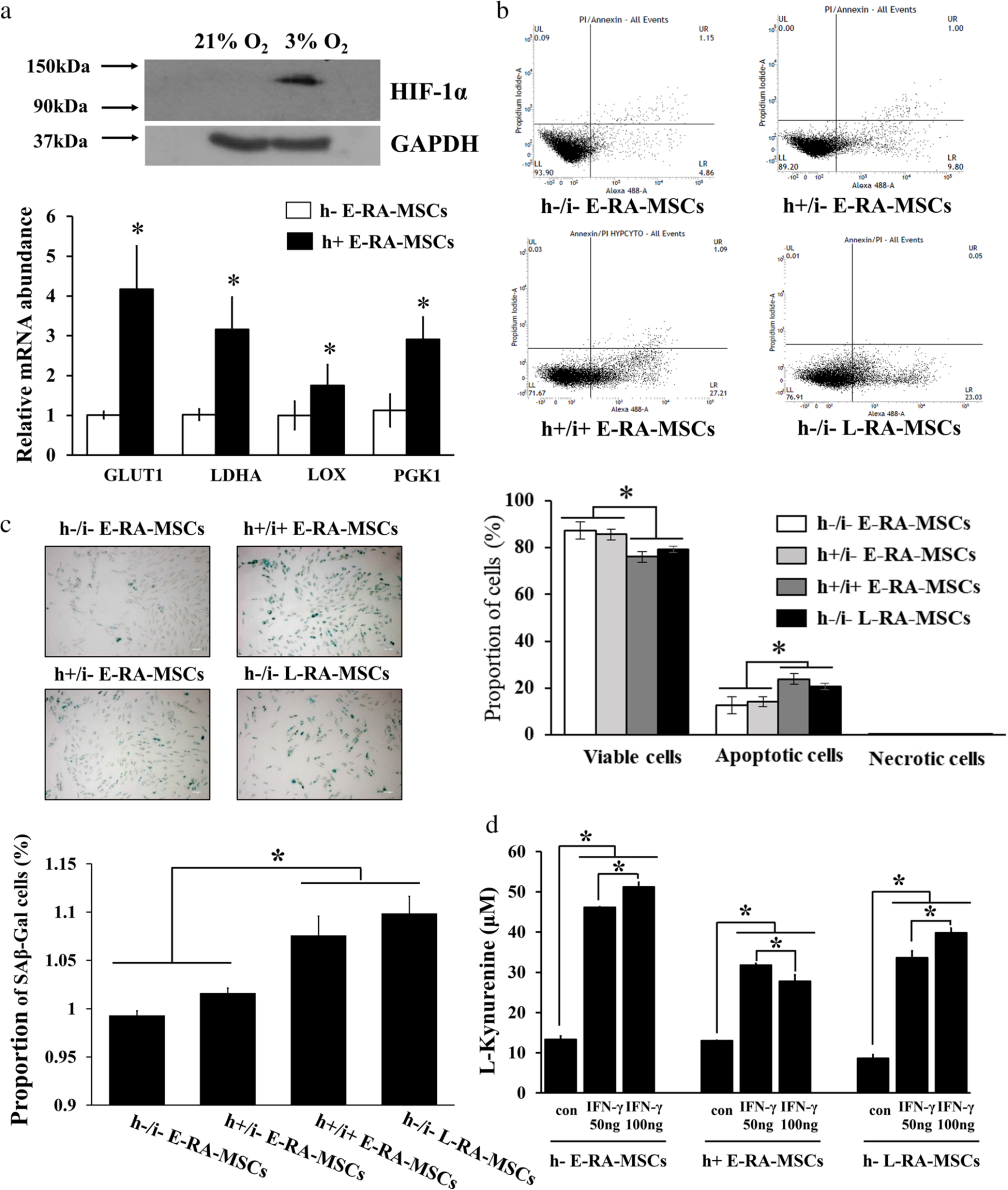
Alteration of SF-MSC senescence by a long-term RA-mimetic milieu. **a** Confirmation of hypoxic culture conditions in E-SF-MSCs by Western blotting and qRT-PCR. 21% O_2_ or 3% O_2_ indicate normoxic or hypoxic culture conditions in SF-MSCs, respectively. **b** Measurement of apoptosis in SF-MSCs with or without a RA joint-mimetic milieu using flow cytometry with Annexin V/PI staining. Cells with Annexin V-/PI-, Annexin V-/PI+ or Annexin V+/PI-, or Annexin V+/PI+ staining were alive, necrotic, or apoptotic, respectively. **c** The range of TRF lengths (kb) in SF-MSCs with or without induced RA joint-mimetic milieu by Southern blotting. **d** Comparison of positive β-gal cell populations in SF-MSCs with or without induction of the RA joint-mimetic milieu. The asterisks indicate significant differences. The data represent the mean ± SD. h- or h+ indicate normoxic or hypoxic conditions, respectively; i- or i+ indicate the absence or presence of pro-inflammatory cytokines, respectively

The synovial tissues of RA patients are exposed to an inflammatory milieu that includes inflammatory cytokines and hypoxia [14]. In particular, L-SF-MSCs derived from the SF of L-RA patients were exposed to prominent pro-inflammatory cytokines for a significantly longer (*P* < 0.05) time than E-SF-MSCs (Figure S1). Therefore, we asked whether an RA joint-mimetic milieu generated by treatment with inflammatory cytokines and hypoxia (h+/i+) could provoke cellular and immunomodulatory alterations in E-SF-MSCs when compared with non-treated L-SF-MSCs (h-/i- L-SF-MSCs). This experiment was performed to clarify the effect of a chronic inflammatory milieu on RA-SF-MSCs. Hypoxia in E-SF-MSCs was validated by significantly increased (*P* < 0.05) HIF-1α levels and the activation of downstream signaling cascades (Fig. 5a). Increased apoptosis and cellular senescence were observed in h+/i+ E-SF-MSCs in the RA joint-mimetic milieu, but not in non-treated E- or hypoxic E-SF-MSCs (h-/i- and h+/i- E-SF-MSCs) (Fig. 5b, c). Although h+/i+ E-SF-MSCs maintained IDO activity and the ability to significantly suppress (*P* < 0.05) PBMC proliferation, the intensities of these activities were weaker than in h+/i- E-SF-MSCs (Fig. 5d). Interestingly, these changes in h+/i+ E-SF-MSCs were similar to those measured in h-/i- L-SF-MSCs, suggesting that the chronic inflammatory milieu of the joints of L-RA patients influences the MSC cellular senescence and immunomodulatory status.

## Discussion

MSCs display a remarkable potential for immunomodulatory properties as a cell-based regimen for inflammatory disease. However, accurate understanding of the therapeutic mechanism of MSCs and their characteristics during disease are particularly important to the development of safe and effective therapeutic strategies. The present study demonstrates the presence of SF-MSC populations in RA patients regardless of disease duration and reveals that immunomodulatory properties of RA-SF-MSCs are attenuated due to inflammation-induced senescence in the inflammatory milieu of RA joints. The immunomodulatory properties of RA-SF-MSCs are especially altered in an RA disease duration-dependent manner. Therefore, our findings suggest that the therapeutic effects of disease-affected MSCs on autoimmune diseases are highly associated with pathology and cellular environmental factors.

**Fig. 6:**
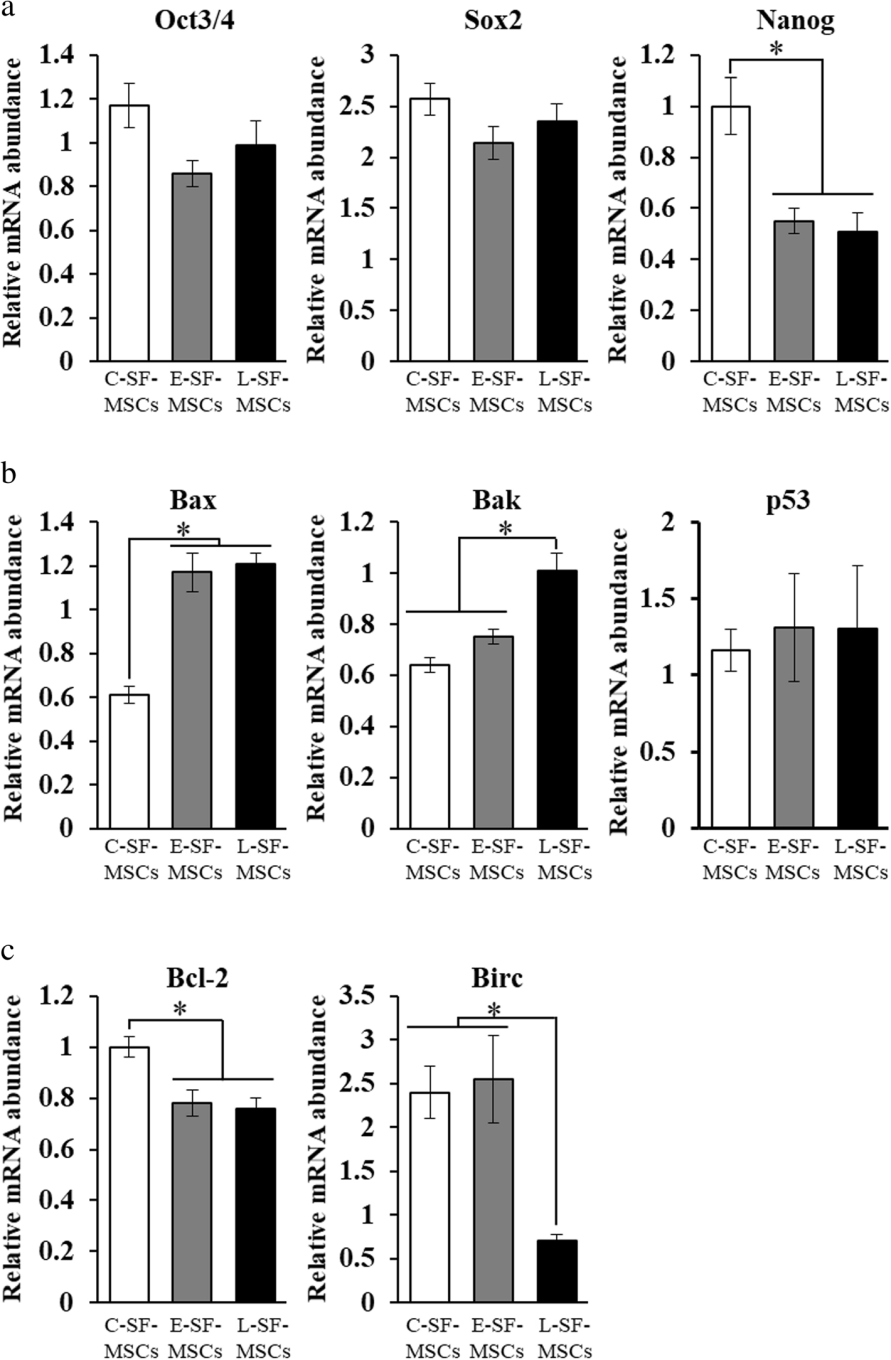
Analysis of gene expression levels in SF-MSCs. Quantitative RT-PCR was used to evaluate **a** pluripotency, **b** pro-apoptosis, and **c** anti-apoptosis gene expression in SF-MSCs. The asterisks indicate significant differences. The data present mean values ± SD

The significant populations of MSCs collected from the SF of arthritic patients have been clearly addressed in previous research. In a study of mesenchymal progenitor cells (MPCs) obtained from RA patients, there were no significant differences between the number of MPCs in the SF from E-RA and L-RA patients, nor in characteristics such as differentiation ability and phenotype [16]. Consistently, the presence of MSCs in SF from RA patients was identified through characterization of the minimal criteria for MSCs, as defined by the International Society of Cellular Therapy [17]. However, our results provide clear evidence for the progression of cellular senescence in RA-SF-MSCs in an RA disease duration-dependent manner, as shown by elevated β-galactosidase-positive cell populations, shortened telomere length, attenuated telomerase activity, apoptosis-related gene expression, and enhanced SASP secretions in L-SF-MSCs exposed to inflammatory cytokines for a longer time compared to CTL- and E-SF-MSCs. As in other autoimmune diseases, MSCs from patients with SLE exhibited increased frequencies of apoptosis, as evidenced by the downregulation of *Bcl-2* and upregulation of *Bax*, and higher intracellular ROS levels than those in normal MSCs [18]. *Bcl-2* is the best-characterized gene of the anti-apoptotic *Bcl-2* family that has been associated with the prevention of cell death initiation [19], and *Birc* is involved in inhibition of apoptosis and cell cycle regulation [20]. By contrast, the pro-apoptotic *Bcl-2* family members *Bax* and *Bak* play apoptosis regulatory functions during development and in tissue homeostasis [21]. The SF-MSCs from RA patients also shared similar alterations, consistent with the results of the present study. Both E-SF-MSCs and L-SF-MSCs showed diminished proliferation ability, with upregulation of pro-apoptotic genes (*Bax* and *Bak*), and downregulation of anti-apoptotic genes (*Bcl-2* and *Birc*), than those of C-SF-MSCs (Fig. 6).

Cellular senescence is activated during intrinsic stress such as extensive cell replication and/or extrinsic stress like ultraviolet radiation, oxidative damage, activated oncogenes, and chronic inflammation. In particular, during the chronic inflammation that occurs with liver cirrhosis, hematopoietic stem cell-associated disorders, chronic human immunodeficiency virus (HIV) infection, myelodysplastic syndromes, ulcerative colitis, and L-RA, immune cells produce strong oxidizing genotoxic substances that induce cellular senescence [22–25]. In the inflammatory milieu, pro-inflammatory cytokines are produced from lymphocytes stimulated by antigens or pathogens. These cytokines activate immune cells, inhibit proliferation of transformed cells, and intensify cellular anti-viral/-tumor effects. Moreover, they induce cellular senescence in various cell types including melanocytes, endothelial cells, and MSCs [10, 25–29]. Cellular senescence is defined as irreversible changes that inhibit cellular division, growth, and function. Senescent MSCs from experimentally induced models, RA patients, and systemic lupus erythematosus (SLE) patients exhibit degradation of distinct cellular features, including differentiation potential, immunomodulatory properties, dysregulated pro-inflammatory cytokine production, and reduced migratory ability [2, 5, 10, 11, 18, 23, 25, 27–31]. Therefore, these studies show that the chronic inflammatory milieu of L-RA is closely associated with cellular senescence in L-SF-MSCs.

Because MSCs modulate responses by both the adaptive and innate immune systems, there is an interest in using MSCs to develop new cell-based approaches for the treatment of various inflammatory diseases [5, 32]. The immunomodulatory properties of MSCs are not spontaneously acquired, but are initiated when MSCs are primed through exposure to pro-inflammatory cytokines and the secretion of anti-inflammatory factors that have inhibitory effects on immune cells [26, 33, 34]. Therefore, administration of MSCs improves the clinical signs associated with autoimmune encephalomyelitis, autoimmune diabetes, multiple sclerosis, polymyositis, atopic dermatitis, and RA [35–37]. Indeed, pre-clinical research of MSCs applications for RA treatment have been actively conducted to clarify the pathogenesis of arthritis and the therapeutic mechanisms of MSCs using CIA and antigen-induced arthritis (AIA) mouse models. MSC-injected CIA or AIA mice display reduced inflammation, ameliorated cartilage destruction, integration of injected MSCs into the synovium, decreased inflammation-induced systemic bone erosion, reduced osteoclast precursors, inhibited receptor activator of NF-κB ligand (RANKL)-induced osteoclastogenesis, elevated anti-inflammatory cytokines, and pro-inflammatory cytokine suppression [3, 38, 39]. However, in the present study, we verified that SF-MSCs do not have the same immunomodulatory properties (Figs. 3 and 4). While CTL-SF-MSCs demonstrated intensive suppression of PBMC proliferation and significant anti-arthritic effects in mice, the immunomodulatory properties of RA-SF-MSCs were attenuated depending on the RA disease status. Moreover, several studies suggest that MSCs are incapable of therapeutically modulating arthritis in CIA mice [6–8]. When considering these and our current results, environmental parameters such as the MSC administration route, the degree of the inflammatory milieu, and/or the condition of the MSCs could influence their immunomodulatory properties. Because the L-SF-MSCs used in the present study were directly exposed in the inflammatory milieu for 13.8 years (Figure S1), we further investigated whether the genotoxic stress caused by chronic inflammation in L-RA patients could alter the characteristics and senescence status of RA-SF-MSCs.

The synovial tissues, including SF, of RA patients are characterized by both chronic inflammation and hypoxic regions compared to synovial tissues in osteoarthritis and healthy patients. The synovial oxygen tension in RA patients was hypoxic from 2 to 4% compared to 9 to 12% at normal [40], and thus, 3% oxygen was exposed as RA hypoxic culture condition (Fig. 5). These symptoms are due to increased oxygen consumption by metabolically active tissue because of the formation of a pannus mass and the recruitment of inflammatory cells. Additionally, newly formed and immature vessels (angiogenesis) cannot supply enough oxygen to the tissue [14, 41–44]. Further, hypoxia can alter mitochondrial activity and increase ROS production and oxidative damage to the inflamed tissues in RA, potentially inducing cell senescence in response to genotoxic substances [42]. Likewise, both hypoxia-conditioned E-SF-MSCs (h+/i- E-SF-MSCs) and non-treated E-SF-MSCs (h-/i- E-SF-MSCs) demonstrated the maintenance of immunomodulatory properties and a non-senescent status. In contrast, E-SF-MSCs in an RA joint-mimetic milieu with hypoxia and pro-inflammatory cytokines (h+/i+ E-SF-MSCs) exhibited senescence-related cellular effects, including increased apoptosis, telomere shortening, elevated β-galactosidase activity, attenuation of IDO activity, and inhibited PBMC proliferation, which were similar to observations made in non-treated L-SF-MSCs (h-/i- L-SF-MSC). Therefore, it is likely that the chronic inflammatory milieu that contains hypoxia and pro-inflammatory cytokines in inflamed RA joints causes genotoxic stress that increases over time, indicating that RA-SF-MSCs have limited immunomodulatory properties at the inflamed site due to inflammation-induced senescence.

## Conclusion

In conclusion, we characterized and investigated how the immunomodulatory properties of RA-SF-MSCs change in response to disease duration. To our knowledge, the present study is the first effort to comparatively evaluate cellular senescence and immunomodulatory characteristics of SF-MSCs under different inflammatory milieus. The three types of SF-MSCs derived from RA patients with different disease prognoses were verified by the differentiation ability and expression of MSC-specific cell surface molecules. Although both E- and L-SF-MSCs were exposed to the inflammatory milieu of RA, the immunomodulatory properties in CIA mice were attenuated depending on disease progression. Cellular senescence was induced by long-term exposure to chronic inflammation and was one reason that L-SF-MSCs had decreased immunomodulatory properties. Therefore, since the fate and potential of MSCs depends on their exposure to the inflammatory environment, the duration of inflammation must be considered prior to the initiation of autologous stem cell applications for inflammatory disease treatment or the clinical application of inflammatory stem cells.

## Supplementary Information

**Additional file 1: Figure S1.** Analysis of cytokine levels in SF. Significant differences between SF-MSCs are indicated by asterisks and the associated *P* values are given above the graphs. The data represent the mean values ± SD.

**Additional file 2: Table S1.** FACS antibodies used in the evaluation of cell surface markers in SF-MSCs.

**Additional file 3: Table S2.** Primer sequences for qRT-PCR.

## Data Availability

Main data have been listed in the primary figures and supplemental data. All the original data are available from the corresponding author on reasonable request.

## Abbreviations

MSCs: Mesenchymal stem cells
RA: Rheumatoid arthritis
SF: Synovial fluid
SF-MSC: Synovial fluid-derived MSC
RA-SF-MSC: SF-MSCs from RA patients
E-SF-MSC: MSC-derived from synovial fluid of early RA patients
L-SF-MSC: MSC-derived from synovial fluid of long-standing RA patients
CTL-SF-MSC: MSC-derived from synovial fluid of healthy controls
CIA: Collagen-induced arthritis
BM-MSC: Bone marrow-derived MSC
ADMEM: Advanced Dulbecco’s modified Eagle’s medium
FBS: Fetal bovine serum
FITC: Fluorescein isothiocyanate
DMEM: Dulbecco’s modified Eagle’s medium
PI: Propidium iodide
TRF: Telomere restriction fragment
PHAL: Phytohemagglutinin-L
BrdU: 5-Bromo-2-deoxyuridine
H&E: Hematoxylin and eosin
TRAP: Tartrate-resistant acid phosphatase
*β*-gal: β-galactosidase activity
SASP: Senescence-associated secretory phenotype
PBMC: Peripheral blood mononuclear cell
IDO: Indoleamine 2,3-dioxygenase
MPCs: Mesenchymal progenitor cells
HIV: Human immunodeficiency virus
SLE: Systemic lupus erythematosus
AIA: Antigen-induced arthritis
RANKL: Receptor activator of NF-κB ligand
TRF: Telomere restriction fragment
RTA: Relative telomerase activity
MMPs: Matrix metalloproteinases family
BS/BV: Bone surface/bone volume
