# Supplementary figures and images for: Chronic inflammation-induced senescence impairs immunomodulatory properties of synovial fluid mesenchymal stem cells in rheumatoid arthritis
Source: Stem Cell Res Ther. 2021 Sep 14;12:502. doi: 10.1186/s13287-021-02453-z (PMC8439066; doi:10.1186/s13287-021-02453-z)

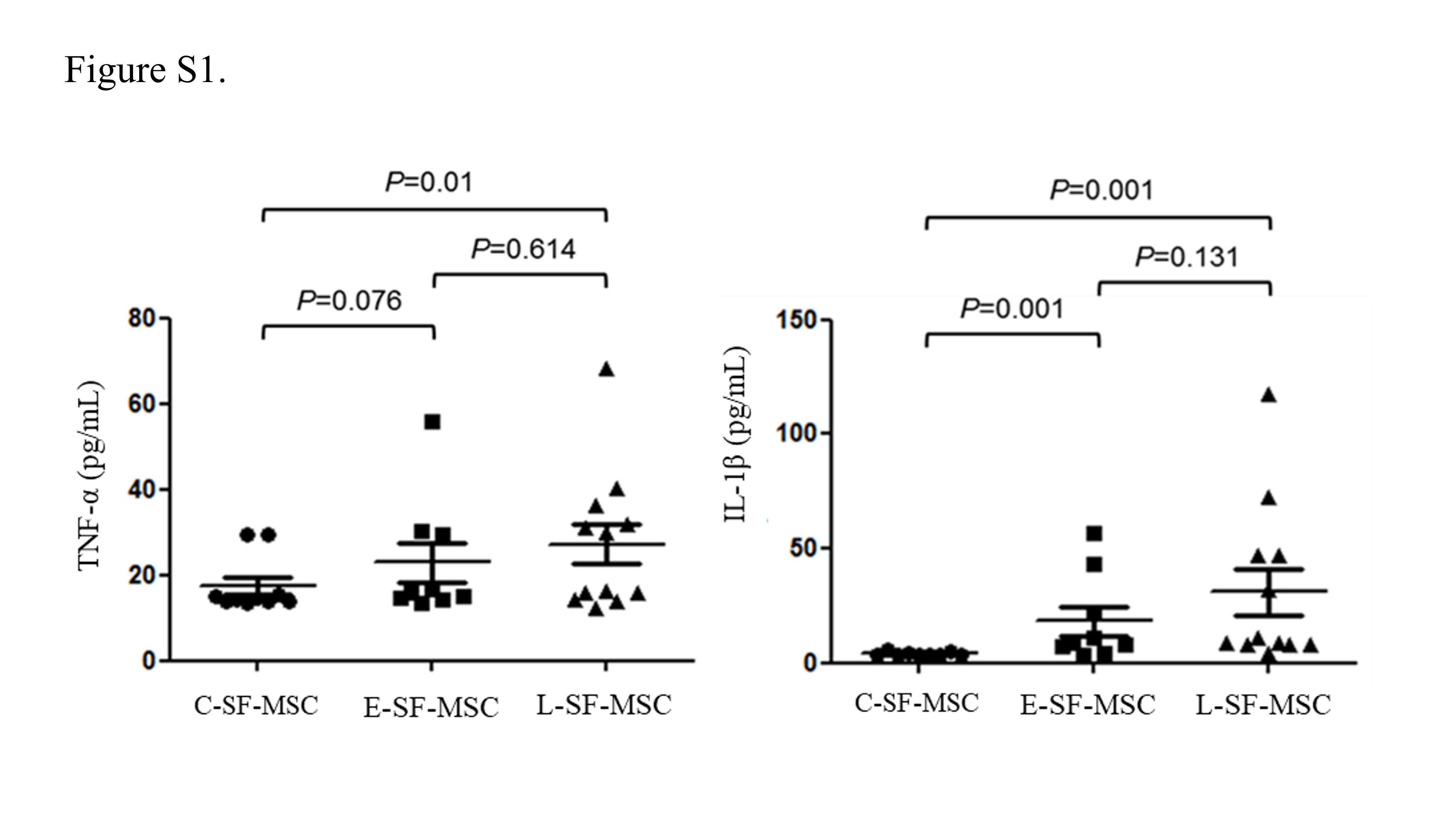

Supplement: Supplementary file 1 — Additional file 1: Figure S1. Analysis of cytokine levels in SF. Significant differences between SF-MSCs are indicated by asterisks and the associated P values are given above the graphs. The data represent the mean values ± SD. [file 13287_2021_2453_MOESM1_ESM.tif]
