# Supplementary material for: Chronic inflammation-induced senescence impairs immunomodulatory properties of synovial fluid mesenchymal stem cells in rheumatoid arthritis
Source: Stem Cell Res Ther. 2021 Sep 14;12:502. doi: 10.1186/s13287-021-02453-z (PMC8439066; doi:10.1186/s13287-021-02453-z)
Supplement: Supplementary file 2 — Additional file 2: Table S1. FACS antibodies used in the evaluation of cell surface markers in SF-MSCs. [file 13287_2021_2453_MOESM2_ESM.docx]

Table S1. FACS antibodies used in the evaluation of cell surface markers in ­­SF-MSCs.

| **Antibody** | **Company** |
| --- | --- |
| FITC goat anti-mouse IgG | BD Pharmingen |
| FITC mouse IgG1, κ isotype control | BD Pharmingen |
| FITC rat anti-mouse CD 44 | BD Pharmingen |
| FITC rat anti-mouse CD90 | BD Pharmingen |
| FITC rat anti-mouse CD 105 | Santa Cruz |
| FITC rat anti-mouse CD 45 | BD Pharmingen |
| Purified mouse anti-human HIF-1α | BD Transduction Laboratories |
| GAPDH rabbit mAb | Cell Signaling Technology |
| Anti-rabbit IgG, HRP conjugate | Promega |
