# Supplementary material for: Chronic inflammation-induced senescence impairs immunomodulatory properties of synovial fluid mesenchymal stem cells in rheumatoid arthritis
Source: Stem Cell Res Ther. 2021 Sep 14;12:502. doi: 10.1186/s13287-021-02453-z (PMC8439066; doi:10.1186/s13287-021-02453-z)
Supplement: Supplementary file 3 — Additional file 3: Table S2. Primer sequences for qRT-PCR. [file 13287_2021_2453_MOESM3_ESM.docx]

Table S2. Primer sequences for qRT-PCR.

| **Gene** | **Primer sequence (5’-3’)** | **GenBank accession number** |
| --- | --- | --- |
| FABP4 | F: GGAAAGTCAAGAGCACCATAACC  R: CATTCCACCACCAGTTTATCATCC | NM_001442.2 |
| PPARγ | F: TACTTTACGCCTCGGTGTTTAGG  R: CCTTCCTTACCTACCACTGACC | NM_001354667.1 |
| ON | F: GTGCAGAGGAAACCGAAGAG  R: AAGTGGCAGGAAGAGTCGAA | NM_003118.2 |
| OCN | F: AGGCACCCTTCTTTCCTCTTCC  R: GGCCCACAGATTCCTCTTCTGG | NM_199173.5 |
| COL2 | F: AGGAATTCGGTGTGGACATAGG  R: GGAAAGTACTTGGGTCCTTTGG | NM_033150.2 |
| COL10A1 | F: AAAGGGACTCATGTTTGGGTAGG  R: AGAGGAGTGGACATACTCAGAGG | NM_000493.3 |
| Oct3/4 | F: ACTATCATTGATGCCCCAGGAC  R: ACACCAGCAGCAACAATCAG | NM_021130.3 |
| Sox2 | F: CACCCACAGCAAATGACAGC  R: AGTCCCCCAAAAAGAAGTCCAG | NM_003106.3 |
| Nanog | F: TGCAACCTGAAGACGTGTG  R: TGGATGGGCATCATGGAAAC | NM_024865.2 |
| Bax | F: TCTGACGGCAACTTCAACTG  R: AGTCCAATGTCCAGCCCATG | NM_138761.3 |
| Bak | F: TATAGACACTTGCTCCCAACCC  R: CTTAGAACCCTCCAGATGAACTCC | NM_001188.3 |
| p53 | F: ACCCAGGTCCAGATGAAG  R: GCAAGAAGCCCAGACGGAAA | NM_000546.5 |
| Bcl2 | F: CTCTGGCAGGCTTAAGATTTGG  R: CAGAGAGGTAAGTGAGCTGTGG | NM_000633.2 |
| Birc | F: CACTTCAGACCCACTTATTTCTGC  R: AGCAGCTTAGATGAGTACAGAGG | NM_001012271.1 |
| GLUT1 | F: GGGAGTGAGACAGAAGTAAGTGG  R: ACTGATGAGAGGTACGTGTAAGG | NM_001012270.1 |
| LDHA | F: GATGTCTTCCTTAGTGTTCCTTGC  R: GTATCTGCACTCTTCTTCAAACGG | NM_006516.2 |
| LOX | F: GCGGTACATATGATCCTTAGCC  R: GCAAAGAGGTACATCAAAGAAGC | NM_001178102.2 |
| PGK1 | F: TTTCTGCATCTCCACTTGGC  R: GATGCTGTGCAACTGTTTAAGG | NM_000291.3 |
| TBP | F: ACGTAATGGCTCTCATGTACCC  R: CAACATCCATCTTCTCACAACACC | NM_001172085.1 |
| ACX | GCGCGGCTTACCCTTACCCTTACCCTAACC | - |
| TS | AATCCGTCGGAGCAGAGTT | - |
